# Impact of Different Land-Use Types on Soil Microbial Carbon Metabolism Function in Arid Region of Alpine Grassland

**DOI:** 10.3390/plants13243531

**Published:** 2024-12-18

**Authors:** Keyi Li, Yaoguang Han, Mo Chen, Guangling Yu, Maidinuer Abulaizi, Yang Hu, Bohao Wang, Zailei Yang, Xinping Zhu, Hongtao Jia

**Affiliations:** 1 College of Bioscience and Resources Environment, Beijing University of Agriculture, Beijing 102206, China; likeyi5771@163.com (K.L.); hyg1968edu@gmail.com (Y.H.); 2 College of Grassland Science, Xinjiang Agricultural University, Urumqi 830052, China; 320200062@xjau.edu.cn (M.C.); mdnr625@163.com (M.A.); 3 College of Resources and Environment, Xinjiang Agricultural University, Urumqi 830052, China; hy@xjau.edu.cn (Y.H.); wangbohao19980922@163.com (B.W.); yzl@xjau.edu.cn (Z.Y.); jht@xjau.edu.cn (H.J.); 4 College of Soil and Water Conservation, Beijing Forestry University, Beijing 100083, China; yuguangling@bjfu.edu.cn; 5 College of Natural Resources and Environment, Northwest Agricultural and Forestry University, Yangling 712100, China; 6 Key Laboratory of Urban Agriculture (North China), Ministry of Agriculture and Rural Affairs, Beijing 102206, China

**Keywords:** land-use types, alpine grassland, Biolog-ECO plate, carbon source utilization, microbial carbon metabolism

## Abstract

There are discrepancies that exist in the effects of different land uses on soil organic carbon (SOC) and soil microbial carbon metabolism functions. However, the impact of land-use type changes on soil microbial carbon metabolism in alpine grassland arid areas is not well understood, hindering our understanding of the carbon cycling processes in these ecosystems. Therefore, we chose three types of land use (continuous reclamation of grassland (RG), abandoned grassland (AG), and natural grazing grassland (GG)) to study the microbial carbon metabolism and its driving factors by the Biolog-ECO method. The results showed that the soil organic carbon content decreased by 16.02% in the RG and by 32.1% in the AG compared to the GG in the 0–20 cm soil layer (*p* < 0.05). Additionally, microorganisms have the highest utilization efficiency of carbohydrate carbon sources, the average values of average well color development (AWCD) were RG (0.26), AG (0.35), and GG (0.26). In the 0–20 cm soil layer, the Shannon–Wiener and the Simpson indices were 3% and 1% higher in the AG compared to the GG, respectively. The soil TOC/TN and soil available phosphorus (AP) were key factors that affected the diversity of soil microbial and carbon metabolism. They were closely related to land-use types. This study holds that abandoning grasslands accelerates the carbon metabolism of microorganisms, leading to the loss of SOC content.

## 1. Introduction

The carbon cycle is the most basic process to maintain ecosystem services. In recent years, land-use change has intensified grassland degradation and desertification worldwide, resulting in a large loss of the grassland carbon pool [1]. The grassland carbon cycle and its response mechanism to land-use change are the scientific bases of grassland protection and sustainable development [2,3]. Soil microbes drive the carbon process by decomposing organic matter, influencing the carbon cycle [4,5,6]. Under varying environmental conditions, the function of carbon cycling in the soil is driven by microorganism changes in response to shifts in the microbial community [7,8,9]. In the global carbon cycle, soil carbon metabolism plays a crucial role and exerts a significant impact on global warming, land-use, and ecological balance [10,11]. Soil microorganisms are a crucial factor in maintaining the material cycle and energy flow of grassland ecosystems. They are sensitive to environmental changes and are good indicators of soil health and grassland degradation [12].

The influence of land-use change on the grassland soil ecosystem has been of wide concern [13,14]. Studies showed that changing land-use types lead to changes in the structure and function of soil microflora [9]. It also had an impact on organic carbon [15]. Grazing activities significantly enhanced the carbon utilization of soil microbial communities, especially for carbohydrate and amino acid carbon sources [16,17,18]. The diversity and composition of microorganisms are essential in determining the functionality and sustainability of grassland soil ecosystems [19]. Biolog-ECO was widely used to evaluate the functional diversity of soil microbial communities under different soil types, different plant species, and land-use types [20,21]. Biolog-ECO quantifies soil microbial carbon utilization and is a simpler method than 16S rRNA gene sequencing, thus effectively and rapidly revealing the impact of environmental change on the functional diversity of microbial communities [22,23]. Research concluded that there were six different carbon sources used by soil microorganisms in different land-use types (taking the alpine grassland in the Sanjiangyuan region as an example). However, they all preferred to use carbohydrate carbon sources [24].

The effects of grazing and fencing were the main focus of research on grassland use on soil microbial diversity and carbon cycling. However, the effects of grassland reclamation and abandonment on soil microbial diversity and carbon cycling were often overlooked, resulting in a lack of clarity on the response of the soil microbial community diversity to reclamation and abandonment in alpine grasslands. This hinders our overall understanding of microbially mediated carbon processes in alpine habitats of different land-use types. Especially, alpine grasslands in arid areas had unique conditions of high altitude, coldness, and aridity, resulting in lower grassland productivity and poor stability, as well as enhanced sensitivity and fragility of the grassland ecosystem.

Therefore, in this study, we assume that the change of grassland utilization mode will change the nutrients in the soil, thus controlling the activity of microorganisms and changing their utilization ability and the diversity of carbon sources. The main objectives of this study are as follows: (1) to analyze the differences of soil microbial diversity and carbon metabolism in different land-use types (reclamation, abandoned cultivation, and natural grassland) and (2) to evaluate the effects of different utilization modes on soil microbial carbon metabolism. These data are an important basis for revealing the transformation of grassland ecological functions under dramatic landscape patterns, and they could provide a theoretical basis for maintaining the function of grassland soil ecosystems and formulating grassland management strategies.

## 2. Materials and Methods

### 2.1. Experimental Site and Sample Land Overview

**Figure 1:**
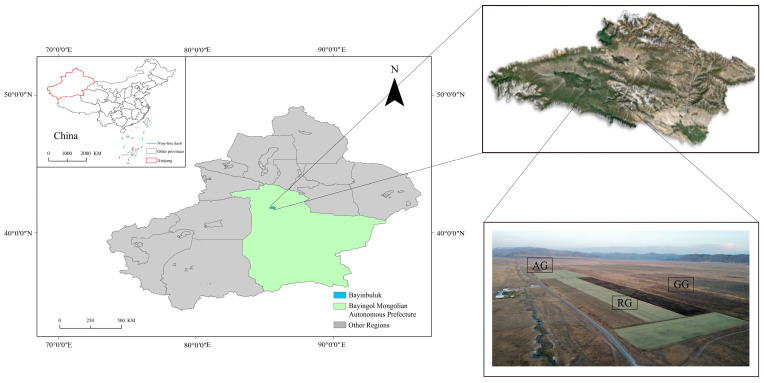
Location of the study area. The location of the study area and the test area in Bayinbuluk alpine grassland. Gray area: Xinjiang Uygur Autonomous Region, China. Continuous reclamation of grassland (RG), abandoned grassland (AG), and natural grazing grassland (GG).

This study was conducted in the Bayinbuluk alpine grassland (42°48.66′–42°49.08′ N, 87°29.10′–84°30.54′ E) in Baxilige village, Hejing County, Xinjiang Uygur Autonomous Region (Figure 1), located in the southern part of the Tianshan Mountains in Central Asia, 2500 m a.s.l. The mean annual temperature is −4.8 °C, with average annual precipitation at 265.7 mm. With long winters and short summers, it belongs to the typical high-altitude climate [25].

Our experiment has three grassland-use types selected: continuous reclamation of grassland (RG), abandoned grassland (AG), and natural grazing grassland (GG). These are summarized as follows: (1) The treatment of RG: in 1974, the alpine grassland was reclaimed to cropland over 48 years of reclamation and planted with *Avena fatua* to the total area of approximately 133 ha. During the planting period, no artificial irrigation or fertilizer was used, and the grass yield was approximately 4500–5250 kg ha^−1^ per year. (2) The treatment of AG: in 1974, the alpine grassland was reclaimed to cropland over 20 years of reclamation and planted with *Avena fatua*; the grasslands cultivated after 1994 were converted into natural pastures. The dominant vegetation is *Elymus dahuricus*, which covers an area of approximately 6 ha. (3) The treatment of GG covers an area of approximately 180 ha. and has been used as a summer pasture since 1974. The vegetation is *Festuca ovina* and *Stipa capillata*. As the annual carrying capacity of grazing pasture is about 0.65 sheep units, it can be considered light grazing.

### 2.2. Soil Sampling

In June 2022, nine sampling points were randomly set for each treatment (RG, AG, and GG). One mixed sample was obtained from each sampling point by the 5-point method, totaling 27 mixed soil samples. And the soil layers of 0–20 cm and 20–40 cm were collected with a shovel. There was a 500 m interval between sample sites. One part of the soil sample was air dried, sieved (1 mm and 0.25 mm), and its soil physical and chemical properties (pH, EC, MC, SOC, TN, TP, TK, AN and AK) were determined. The other part of the soil sample was stored in a 4 °C refrigerator and sieved with a 2 mm sieve screen for soil the Biolog-ECO test.

### 2.3. Soil Physical and Chemical Properties Measured

Soil pH and soil electric conductivity (EC) were determined using a pH/EC meter (water/soil ratio 5:1; Mettler Toledo FE28-Standard, Switzerland) [26]. Soil organic carbon (SOC) content was determined by the H_2_SO_4_-K_2_Cr_2_O_7_ external heating method [12]. The determination of organic carbon in our test (H_2_SO_4_-K_2_Cr_2_O_7_) can only oxidize 90% of organic carbon; thus, we multiply by the correction coefficient of 1.1 to calculate the content of organic carbon [27]. Soil alkali hydrolyzed nitrogen (AN) was determined by the alkali-hydrolyzed diffusion method. Soil total nitrogen (TN) was determined by an elemental analyzer (Euro-EA3000, Eurovector, Pavia, Italy) [28]. The total phosphorus (TP) in the soil was determined by the acid digestion molybdenum-antimony colorimetric method (Shimadzu UV-1780, Shimadzu, Kyoto, Japan), and the recovery rate of TP was 95–104% [29,30]. The available phosphorus (AP) in soil was determined by the sodium bicarbonate extraction molybdenum-antimony colorimetric method (Shimadzu UV-1780, Shimadzu, Kyoto, Japan), and the recovery rate of AP was 100.63–118.84% [30,31]. Soil available potassium (AK) was extracted by CH_3_COONH_4_, and total potassium (TK) was boiled by H_2_SO_4_-HClO_4_ [28]. The contents of soil AK and TK were determined by a flame photometer (M420, Sherwood, Nottingham, UK). Soil moisture content was measured by the drying method: 10 g of fresh soil was put into an aluminum box for drying (105 °C for 24 h), and its dry weight was measured, and soil moisture content (MC) was obtained by calculation [32].

### 2.4. Biolog-ECO Test

A mixture of 10 g of fresh soil sample and 95 mL of sterile 0.145 mol L^−1^ NaCl solution was shaken on a shaking table for 15 min. After the oscillation was completed, 2 mL of supernatant was added into a Petri dish containing 18 mL 0.85% NaCl solution on a super-clean work table, and the sample liquid was absorbed repeatedly to clean the gun head and mixed well. After dilution, a 125 µL dilution suspension was injected into each hole of the Biolog-ECO plate, which has 96 micro-wells with 32 wells per replicate, including 31 carbon sources and 1 blank, and 3 replicates in total were used in this study (Table 1). Three Biolog-ECO plates were used per sample for a total of 9 replicates. These were cultured in an incubator at 25 °C for 144 h, detected every 12 h at 590 nm with an enzyme labeler (Multiskan SkyHigh, Thermo Fisher Scientific, Waltham, MA, USA), and the color changes were recorded [33,34].

The culture value of 12–120 h average well color development (AWCD) increased rapidly, the microorganisms entered the exponential growth period, the substrate carbon source was consumed greatly, and entered the stable period after 120 h. Therefore, we observed the AWCD for 120 h. The AWCD Biolog-ECO data after 120 h of incubation were analyzed as follows: (1) AWCD of a single hole [33]: $AWCD=\sum \left(C_{i}-R_{i}\right)/n$. Ci represents the absorbance of each hole, Ri represents the absorbance of the control hole, and n represents the number of carbon sources. (2) The Shannon–Wiener index (H’) was used to estimate species richness [35]: $H^{\prime }=-\sum P_{i}(\ln P_{i})$. In the formula, $P_{i}=\left(C_{i}{-R}_{i}\right)/\sum \left(C_{i}-R_{i}\right)$. (3) The Simpson index (D) [36] was used to assess the dominance of certain species $D=1-\sum P_{i}^{2}$. (4) Pielou evenness index (E) [24]: $E=H^{\prime }/\ln S$, where S denotes the number of carbon sources employed.

### 2.5. Data Analysis

Two-factor ANOVA and Spearman correlation analysis of soil physical and chemical properties were analyzed using the SPSS software (IBM Corporation, 21st edition, located in New York City, NY, USA). To examine the association between soil microbiota carbon sources and physical and chemical properties, redundancy analysis (RDA) was employed as a statistical method. RDA allowed for the exploration and interpretation of the relationship between these variables. The Origin Pro 8. and R language were used for data visualization.

Partial Least Squares Path Modeling (PLS-PM) is a widely utilized methodology for the study of complex relationships between multiple variables as a statistical analysis technique. It combines the strengths of path analysis and factor analysis and can help to reveal correlations between the direct and indirect effects of variables [37]. PLS-PM, using the “plspm” package in R (version 4.1.2), was utilized to further determine the relationships between land-use types, microbial diversity, capacity to utilize carbon sources, and environmental variables. To evaluate the usability of the PLS-PM method, non-parametric test measures were used, including commonality, the mean coefficient of determination (R^2^) of all latent variables, the normalized root-mean-square residual (SRMR), and the goodness of fit index (GFI) [38]. Carbon utilization capacity and microbial diversity are two potential factors represented by carbohydrates,, amino acids, carboxylic acids, polymers, phenolic acids, and amines, as well as the Shannon–Wiener index, Simpson index, and Pielou evenness index. Path coefficients represent the direction and strength of the linear relationship between variables, thus representing direct effects. Indirect effects, on the other hand, are the product of the path coefficients obtained by using indirect paths [39]. In addition, the GFI was used to estimate the predictive performance of the model. And the correlation between microbial carbon limit and other variables was determined using the “vegan” and “ggplot 2” packages for multivariate analysis. Linear regression for the entire test was assumed to be fitted by SPSS (linear).

## 3. Results

### 3.1. Soil Physical and Chemical Properties Under Different Land-Use Types

The soil physical and chemical properties were significantly different among the three treatments in the 0–20 cm and 20–40 cm soil layers (Table 2). The soil moisture content (MC) was significantly higher in the RG compared to both the AG and GG in the 0–20 cm soil layer (*p* < 0.05). The soil TK content was significantly increased in the RG compared to the GG in the 0–20 cm soil layer. However, the soil TK content in the AG showed a significant decrease (*p* < 0.05). The SOC content in the RG and AG were significantly decreased by 16.02% and 32.1% compared with the GG in the 0–20 cm soil layer (*p* < 0.05). Furthermore, compared to the GG, the soil AP content in the RG and the AG were significantly decreased by 17.29% and 46.01% in the 0–20 cm soil layer (*p* < 0.05). And compared to the GG, the AK content was significantly decreased by 35.03% and 28.51% in the RG and the AG in the 0–20 cm soil layer (*p* < 0.05). The contents of SOC, TK, and TP in the RG and the AG were significantly decreased compared with the GG in the 20–40 cm soil layer (*p* < 0.05). And the contents of SOC in the RG and the AG were decreased by 10.59% and 28.16% in the 20–40 cm soil layer (*p* < 0.05). Furthermore, the RG and the AG showed a significant increase in ammonium nitrogen (AN) content by 32.25% and 11.83% compared with the GG in the 20–40 cm soil layer (*p* < 0.05). However, the AP content in the RG and the AG were significantly decreased by 65.50% and 24.72% compared with the GG in the 20–40 cm soil layer (*p* < 0.05). The trend of the TOC/TN ratio was consistent with SOC. And the two-way ANOVA of soil physical and chemical properties indicated that there was an interaction effect of soil layer and land-use type on all indicators with significant responses (Table 3). Except TN, the effects of different land-use types on other soil physical and chemical properties are less than those of soil layers.

### 3.2. Carbon Utilization Capacity of Microorganisms Under Different Land-Use Types

**Figure 2:**
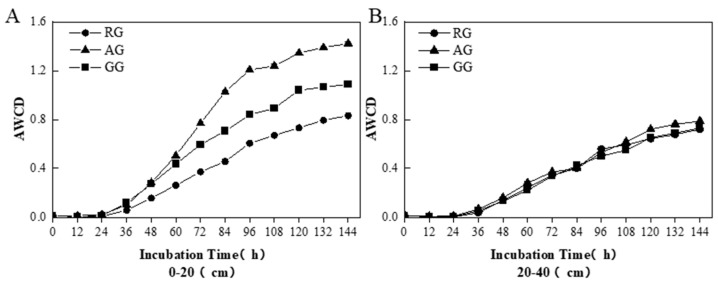
Changes in AWCD during soil microbial community cultivation in grasslands under land-use type. (**A**) shows the change in AWCD values from 0 to 144 h in the 0–20 cm soil layer. (**B**) shows the change in AWCD values from 0 to 144 h in the 20–40 cm soil layer. Continuous reclamation of grassland (RG), abandoned grassland (AG), and natural grazing grassland (GG).

**Figure 3:**
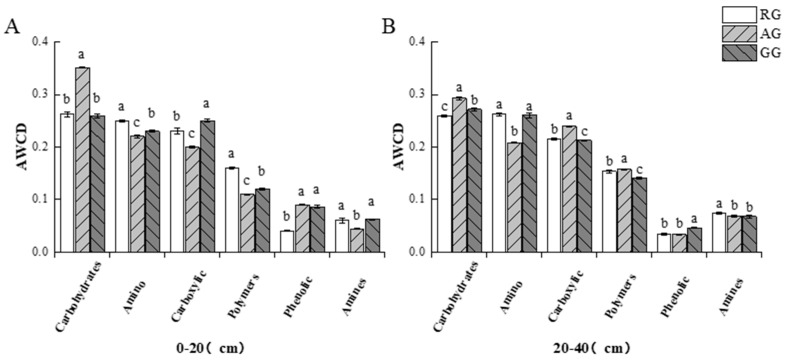
Utilization rate of six carbon sources of soil microorganisms in different treatments of grasslands. (**A**) shows the utilization of six carbon sources of soil microorganisms in the 0–20 cm soil layer. (**B**) shows the utilization of six carbon sources of soil microorganisms in the 20–40 cm soil layer. Different superscript lowercase letters indicate significant differences in different processing data, *p* < 0.05. Continuous reclamation of grassland (RG), abandoned grassland (AG), and natural grazing grassland (GG).

The AWCD showed significant variations in the 0–20 cm soil layer among the three treatments (Figure 2). Compared with the GG, AWCD of the AG showed a significant increase and indicated the stronger utilization ability of the soil microbial community to carbon sources and higher physiological and metabolic activities (Figure 2A). However, compared to the GG, the AWCD of the RG was significantly lower (*p* < 0.05), indicating that the soil microbial community had a lower capacity to utilize carbon sources and lower carbon metabolism activities. The AWCDs among the three treatments were not significantly different from each other in the 20–40 cm soil layer (Figure 2B). The absorbance value of 120 h was used to analyze the utilization rate of six types of carbon sources by different soil microbial communities (Figure 3). The utilization rate of carbohydrate carbon source for RG was 0.35, which was significantly higher than AG and GG (*p* < 0.05). The ability to utilize microbial carbon sources of the AG was in the order of carbohydrates, amino acids, carboxylic acids, polymers, phenolic acids, and amines in the 0–20 cm soil layer. And amine carbon source utilization in the RG was 0.04, which was significantly lower than the AG and the GG (*p* < 0.05) (Figure 3A). However phenolic acid carbon source utilization in the RG and the AG was 0.03, which was significantly lower than the AG and the GG in the 20–40 cm soil layer (*p* < 0.05) (Figure 3B).

### 3.3. Carbon Metabolism Function and Diversity of Soil Microbial Community Under Different Land-Use Types

**Figure 4:**
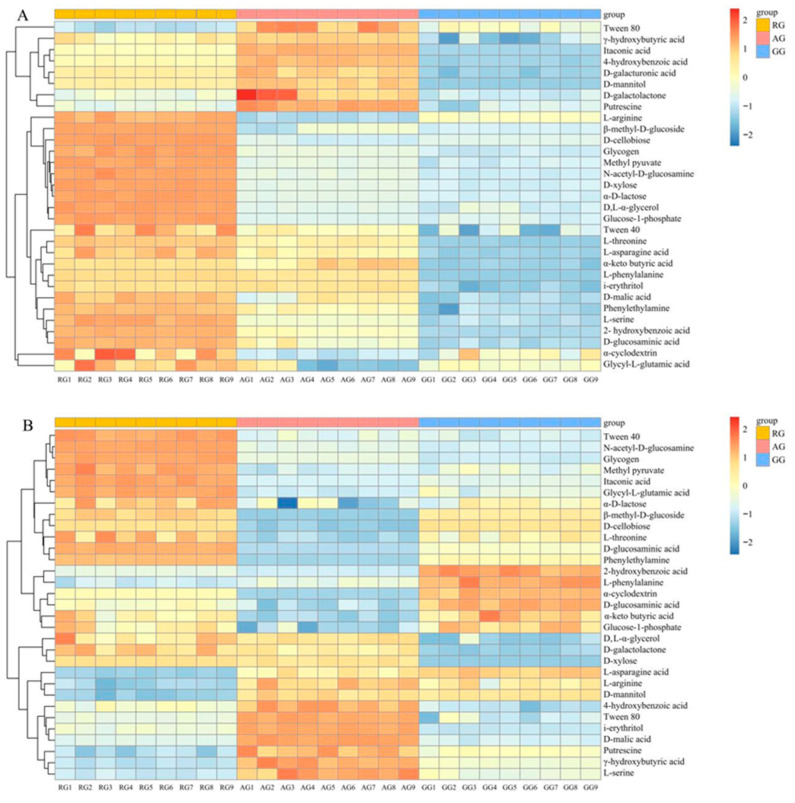
Heat map of functional carbon metabolism of soil microorganisms. (**A**) shows the thermogram of soil microbial functional carbon metabolism in the 0–20 cm soil layer. (**B**) shows the thermogram of soil microbial functional carbon metabolism in the 20–40 cm soil layer. RG 1–9 represents the nine samples treated with RG. AG 1–9 represents the nine samples treated with AG. GG 1–9 represents the nine samples treated with GG. Continuous reclamation of grassland (RG), abandoned grassland (AG), and natural grazing grassland (GG).

RG was significantly enriched for functional microorganisms with *L-asparagine*, *D-xylose*, *L-serine*, *β-methyl-D-glucoside*, *i-erythritol*, etc., as carbon sources in the 0–20 cm soil layer (Figure 4A). However, RG was significantly enriched for functional microorganisms with *Tween 40, a-D-lactose, D-galacturonic acid, Glycogen, L-threonine*, etc., as carbon sources in the 20–40 cm soil layer (Figure 4B). AG was significantly enriched for functional microorganisms with *Tween 80, γ-hydroxybutyric acid, D-mannitol, 4-hydroxybenzoic acid, Putrescine*, etc., as carbon sources in the 0–40 cm soil layer (Figure 4). None of the functional microorganisms from the carbon sources were significantly enriched for the GG in the 0–20 cm soil layer. However, *D-glucosaminic acid*, *2-hydroxybenzoic acid*, *L-phenylalanine*, and *α-cyclodextrin* were significantly enriched for the GG in the 20–40 cm soil layer. In summary, microbial communities with different soil layers and different treatments had various carbon source utilization capacities.

**Figure 5:**
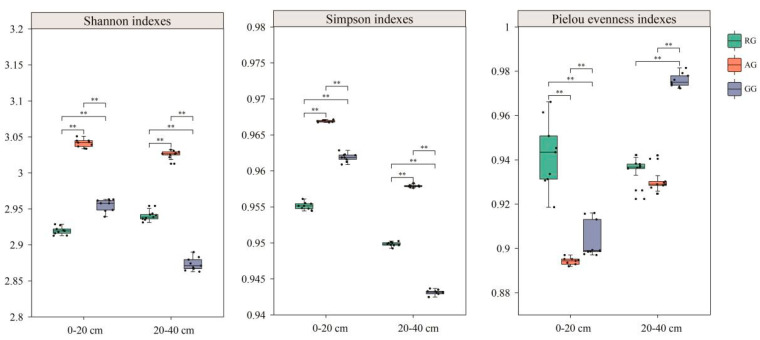
Index of soil microbial community functional diversity for three grassland treatments. ** *p* < 0.01. Continuous reclamation of grassland (RG), abandoned grassland (AG), and natural grazing grassland (GG).

Compared to the GG, the Shannon–Wiener and Simpson indices of the RG were significantly lower, while the Shannon–Wiener and Simpson indices of the AG showed a significant increase in the 0–20 cm soil layer (*p* < 0.05) (Figure 5). However, compared with the GG, both the Shannon–Wiener and Simpson indices were significantly increased in the RG and the AG in the 20–40 cm soil layer. And the Pielou evenness index in the RG and the AG showed a significant decrease compared with the GG (*p* < 0.05). It can be concluded that the AG exhibits more microorganism functional diversity in soil microbial communities than the RG and the GG.

### 3.4. Relationship Between Metabolic Function of Soil Microbial Community and Soil Environmental Factors Under Different Utilization Types

**Figure 6:**
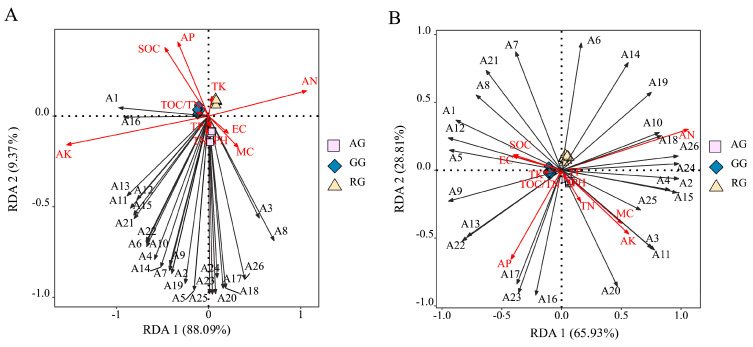
RDA analysis of soil physicochemical properties and soil carbon metabolism. (**A**). RDA analysis of soil physicochemical properties and soil carbon metabolism in the 0–20 cm soil layer. (**B**). RDA analysis of soil physicochemical properties and soil carbon metabolism in the 20–40 cm soil layer. A1, *Putrescine*; A2, *Phenylethylamine*; A3, *Glycyl-L-glutamic acid*; A4, *L-threonine*; A5, *L-serine*; A6, *L-phenylalanine*; A7, *L-asparagine acid*; A8, *L-arginine*; A9, *D-malic acid*; A10, *α-ketobutyric acid*; A11, *Itaconic acid*; A12, *γ-hydroxybutyric acid*; A13, *4-hydroxybenzoic acid*; A14, *2-hydroxybenzoic acid*; A15, *D-galacturonic acid*; A16, *D-galactolactone*; A17, *D,L-α-glycerol*; A18, *Glucose-1-phosphate*; A19, *D-glucosaminic acid*; A20, *N-acetyl-D-glucosamine*; A21 *D-mannitol*; A22, *i-erythritol*; A23, *D-xylose*; A24, *β-methyl-D-glucoside*; A25, *α-D-lactose*; A26, *D-cellobiose*; A27, *Glycogen*; A28, *α-cyclodextrin*; A29, *Tween 80*; A30, *Tween 40*; A31, *Methyl pyruvate*. MC: soil moisture content; SOC: soil organic carbon; TN: soil total nitrogen; TP: soil total phosphorus; TK: soil total potassium; AN: soil available nitrogen; AP: soil available phosphorus; AK: soil available potassium. Continuous reclamation of grassland (RG), abandoned grassland (AG), and natural grazing grassland (GG).

*D-galactolactone* was the main source of microbial carbon utilization in the GG. It was significantly positively correlated with AK and SOC but negatively with AN in the 0–20 cm soil layer (Figure 6A). For the AG, *D-xylos* and *β-methyl-D-glucoside* were the main sources of microbial carbon utilization, and they were significantly negatively correlated with AK in the 0–20 cm soil layer. However, for the AG, *Glycyl-L-glutamic acid* and *Itaconic acid* were the main sources of microbial carbon utilization, and they were significantly and positively associated with AK in the 20–40 cm soil layer (Figure 6B). And for the RG, *L-phenylalanine* was the main source of microbial carbon utilization in the 20–40 cm soil layer. It was significantly negatively associated with AK and AP in the 20–40 cm soil layer (Figure 6B).

**Figure 7:**
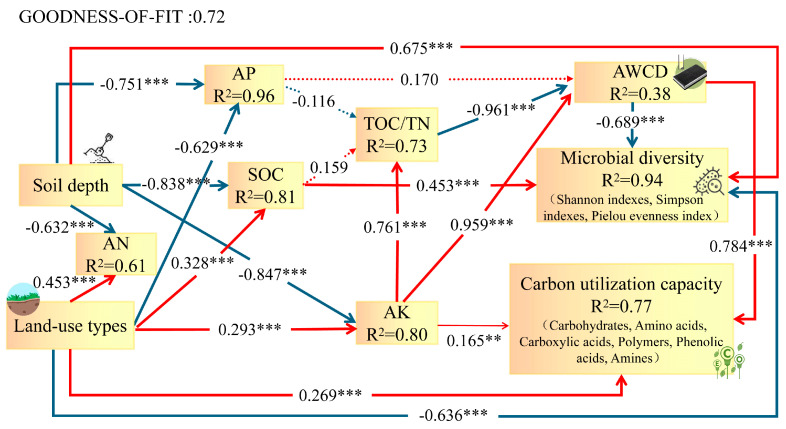
PLS-PM analysis of microbial carbon metabolism function and soil layer with different land-use types. The red and blue arrows represent the positive and negative effects, respectively. The arrow line width reflects the size of the path coefficient. Numbers represent the path coefficient (direct effect), representing the strength and direction of the relationship between the variables. *** represents the statistical significance at *p* < 0.001; ** represents the statistical significance at *p* < 0.01. The proportion of variance (R^2^) is shown below each variable in the model. SOC: soil organic carbon; AN: soil available nitrogen; AP: soil available phosphorus; AK: soil available potassium; AWCD: average well color development. Microbial diversity includes the Shannon index, Simpson index, and Pielou evenness index. Carbon utilization capacity includes carbohydrates, amino acids, carboxylic acids, polymers, phenolic acids, and amines.

The PLS-PM method was used to analyze the relationship between microbial carbon metabolism function in different land-use types and soil layer (Figure 7). The results show that different land-use patterns have a direct positive impact on AK and an indirect positive impact on AWCD. However, AK has a positive direct effect on TOC/TN and an indirect negative effect on AWCD. At the same time, the utilization mode has a direct positive impact on carbon utilization capacity and a direct negative impact on microbial diversity. In addition, soil layer also directly and positively affects microbial diversity.

## 4. Discussion

### 4.1. Effects of Different Land-Use Types on Soil Physical and Chemical Properties

Different land-use types, including reclamation, abandonment, and grazing, significantly influence soil structure and nutrient cycling in grasslands [40,41]. In grassland ecosystems, organic carbon primarily exists in the form of organic matter, predominantly concentrated in the top 0–20 cm layer of soil. The organic carbon content and physical and chemical properties of abandoned grasslands were found to be lower, which contradicts the findings of Nautiyal [42]. This discrepancy may be attributed to the lack of fertilization in abandoned grasslands, which are often not supported by effective soil conservation measures, making them more vulnerable to soil erosion. Soil erosion can lead to the loss of organic matter, thereby reducing both the quantity and quality of organic carbon in the soil. Additionally, decreased soil biodiversity may result in fewer organic carbon sources, further diminishing the soil’s organic carbon content [28]. Our study found that total nitrogen (TN) content in abandoned grasslands (AG) was significantly higher than in other treatments. At the same time, the ratio of C/N in this area shows that the plant community of AG may have higher nitrogen content in its tissues. This increase can be attributed to the fact that the years of recovery after fallow or abandonment often allow the formation of specialized plant communities distinct from reclaimed or natural grasslands [43]. The root systems of these plant communities may be more developed and more prone to fix nitrogen into the soil. Moreover, abandoned grasslands may have experienced longer recuperation, giving plants longer growth cycles and enhanced nitrogen accumulation [43]. Additionally, the pH of GG was lower than that of RG and AG, likely due to the deposition of organic matter from cattle and sheep feces, which contributes to lower pH levels. This pH variation is also influenced by multiple factors, including vegetation cover, organic carbon content, and agricultural land management practices [44].

### 4.2. Effect of Different Land-Use Types on Carbon Metabolism Function and Diversity in Soil Microbial Communities

Soil microorganisms play a crucial role in soil energy flow and material cycling [45]. The method employed in this study primarily captured the microbial information relevant to carbon source utilization on the Biolog-ECO plate. Consequently, the types of carbon metabolic diversity obtained experimentally inadequately represented the functional diversity of the entire soil microbial community. The AWCD metric effectively reflects the overall carbon utilization status of microbial communities, demonstrating its utility in assessing the capacity of soil microbes to utilize various carbon sources [44,46]. The results revealed that abandoned grasslands with higher AWCD exhibited elevated utilization of carbohydrate carbon sources, aligning with the findings of Paixao et al. and Zhang et al. [34,47]. This is because grasslands typically exhibit good nutrient coverage, with carbohydrates secreted by plants and root residues providing essential carbon for soil microorganisms [48].

Most research indicates that grassland reclamation tends to reduce soil microbial activity and diversity [49]. In this study, both the Shannon–Wiener and Simpson indices were found to be higher in abandoned grasslands compared to continuously reclaimed and naturally grazed grasslands in the 0–20 cm soil layer. The increase in plant litter and organic matter in the soil, compared to reclaimed grasslands, stimulates the carbon metabolic functions of soil microorganisms [50]. However, no significant differences were observed in the soil microbial carbon utilization capacity among the three land-use treatments in the 20–40 cm soil layer. This is likely because the 0–20 cm layer contained a higher concentration of roots, and different land-use types supported varying vegetation and root systems [51,52]. Furthermore, well-developed and dense plant roots at the surface contribute significant exudates and plant litter, serving as primary energy sources for microorganisms [53,54]. Additionally, grazing in abandoned grasslands, along with the addition of organic matter from cow and sheep dung, also influences microbial activity in the topsoil layer [16,28]. In addition, land cultivation may lead to soil structure degradation and disruption of the carbon cycle, thereby negatively impacting the carbon metabolic capacity and microbial diversity of the soil in the study area [26].

### 4.3. Relationship Between Metabolic Functions of Soil Microbial Communities and Soil Environmental Factors

Different land-use types significantly influence soil microbial diversity and the surrounding soil environment, revealing a complex interplay between these elements [55]. This aligns with findings from related studies, which suggest that the interaction between soil microbial communities and variations in SOC and total nitrogen (TN) mirrors the results observed in this research [56,57]. Furthermore, carbohydrates and phenolic acid compounds play a crucial role in decomposing plant litter on the soil surface. This decomposition process alters the physical and chemical characteristics of the rhizosphere soil, leading to significant impacts on both the species composition and abundance of rhizosphere microorganisms [58]. Such alterations in the rhizosphere environment are likely the primary reason for the pronounced differentiation observed in the metabolic characteristics of soil microbial communities, particularly regarding their carbohydrate utilization capabilities. Interestingly, while it is acknowledged that different land-use types indirectly influence the microbial carbon source utilization capacity through variations in SOC content. This study found that the diversity of soil microorganisms was closely related to nitrogen and phosphorus. This finding contrasts with earlier studies that emphasized organic carbon content as the principal influencing factor [16,59]. Grassland land-use modifications might have prompted vegetation types and soil organic matter changes, consequently altering the C, N, and *p* content in their compositions [43]. In addition, various land-use types indirectly enhance microbial diversity by improving soil nutrient content. This relationship underscores the multifaceted effects that land management practices can have on microbial communities and their functioning. In conclusion, this study highlights that land-use types exert both direct and indirect influences on the average well color development (AWCD) of soil microorganisms by regulating AN, available phosphorus (AP), and SOC. This may be because the supply of N and P directly affects the activity of soil microorganisms and the mineralization process of carbon [60]. And microbial metabolic efficiency may also play an important role in controlling the C-N interaction. Microbial metabolic efficiency usually increases with the increase in N availability, and this physiological characteristic adjustment can promote the biomass C/N to meet its nitrogen demand and maintain balance [61]. Some studies also show that the main driving factors of soil microbial nutrient metabolism limitation include soil total nutrients, C/N, and available nutrients [62]. Such interactions culminate in a comprehensive effect on the microbial carbon metabolism capacity. By promoting vegetation restoration in grassland ecosystems through fertilization or reseeding, as well as improving soil fertility (AN, AP, and SOC), not only can the carbon utilization capacity of soil microorganisms be enhanced but also the metabolic activity and functional diversity of the soil microbial community can be improved [63].

## 5. Conclusions

Our study assessed microbial carbon metabolism in grassland ecosystems, providing valuable insights into the factors that influence changes in land-use types. The findings revealed that different land-use types significantly impacted the physical and chemical properties of the soil. The soil microbial carbon metabolic capacity in AG is significantly higher than that in RG and GG. This paper shows that the microbial carbon metabolism capacity is enhanced during the recovery of abandoned grassland. And the richness of soil microorganisms is also significantly improved. Furthermore, the key factors such as available nutrients (AN, AP, and AK) and TOC/TN were identified as critical, affecting soil microbial carbon metabolism and microbial activity. Notably, the change of utilization mode promotes microbial carbon metabolism, but inhibits microbial diversity. Additionally, the influence of land-use types on the ability of microorganisms to utilize carbon sources was more pronounced in the 0–20 cm soil layer compared to the 20–40 cm layer. To promote grassland microbial diversity and facilitate abandoned grassland natural restoration, appropriate restoration measures (such as fertilization, reseeding, and fencing) should be implemented. In future studies, the impact of vegetation restoration in abandoned grasslands on soil microbial diversity can be further explored. This approach will also aid in better regulating and managing the carbon cycle across grassland ecosystems with varying land-use types.

## Figures and Tables

**Table 1:** Six types of carbon sources.

Types	Carbohydrates	Amino Acids	Carboxylic Acids	Polymers	Phenolic Acids	Amines
Carbon sources	*D-cellobiose*	*L-arginine*	*Methyl pyruvate*	*Tween 40*	*2-hydroxybenzoic acid*	*Phenylethylamine*
	*α-D-lactose*	*L-asparagine acid*	*D-galacturonic acid*	*Tween 80*	*4-hydroxybenzoic acid*	*Putrescine*
	*β-methyl-D-glucoside*	*L-phenylalanine*	*γ-hydroxybutyric acid*	*α-cyclodextrin*		
	*D-xylose*	*L-serine*	*D-glucosaminic acid*	*Glycogen*		
	*i-erythritol*	*L-threonine*	*Itaconic acid*			
	*D-mannitol*	*Glycyl-L-glutamic acid*	*α-keto butyric acid*			
	*N-acetyl-D-glucosamine*		*D-malic acid*			
	*D, L-α-glycerol*					
	*Glucose-1-phosphate*					
	*D-galactolactone*					

**Table 2:** Soil physical and chemical properties.

Soil Depth (cm)	Land-Use Types	pH	EC (mS·cm−3)	MC (%)	SOC (g·kg−1)	TN (g·kg−1)	TP (g·kg−1)	TK (g·kg−1)	AN (mg·kg^−1^)	AP (mg·kg−1)	AK (mg·kg−1)	TOC/TN
0–20	RG	7.90 ± 0.03 ^a^	1534.61 ± 18.01 ^a^	8.75 ± 0.16 ^a^	37.80 ± 0.61 ^b^	2.19 ± 0.17 ^c^	2.96 ± 0.05 ^b^	17.77 ± 0.44 ^a^	177.6 ± 5.81 ^a^	11.29 ± 0.59 ^b^	109.56 ± 1.66 ^b^	2.12 ± 0.08 ^b^
	AG	7.88 ± 0.04 ^a^	1421.94 ± 22.51 ^c^	8.95 ± 0.25 ^a^	30.56 ± 0.77 ^c^	2.60 ± 0.40 ^a^	3.05 ± 0.03 ^a^	14.91 ± 0.14 ^c^	147.4 ± 1.08 ^b^	7.37 ± 0.51 ^c^	120.56 ± 4.44 ^b^	2.13 ± 0.28 ^b^
	GG	7.69 ± 0.04 ^b^	1475.67 ± 25.40 ^b^	6.05 ± 0.26 ^b^	45.01 ± 0.74 ^a^	2.36 ± 0.47 ^b^	2.89 ± 0.04 ^c^	16.43 ± 0.30 ^b^	128.52 ± 3.05 ^c^	13.65 ± 0.70 ^a^	168.64 ± 5.52 ^a^	2.72 ± 0.06 ^a^
20–40	RG	8.24 ± 0.02 ^a^	1319.56 ± 17.25 ^a^	10.17 ± 0.19 ^b^	30.23 ± 1.23 ^b^	1.85 ± 0.26 ^b^	2.85 ± 0.04 ^ab^	15.59 ± 0.37 ^b^	129.09 ± 3.87 ^a^	1.87 ± 0.50 ^c^	76.51 ± 2.04 ^a^	1.94 ± 0.08 ^b^
	AG	8.06 ± 0.03 ^c^	1149.67 ± 23.50 ^b^	12.95 ± 0.24 ^a^	24.29 ± 0.61 ^c^	2.55 ± 0.38 ^a^	2.82 ± 0.03 ^b^	13.66 ± 0.28 ^c^	109.16 ± 1.47 ^b^	4.08 ± 0.59 ^b^	78.93 ± 3.12 ^a^	1.78 ± 0.04 ^c^
	GG	8.17 ± 0.03 ^b^	1329.33 ± 20.35 ^a^	8.69 ± 0.23 ^c^	33.81 ± 0.41 ^a^	1.88 ± 0.27 ^b^	2.86 ± 0.04 ^a^	16.80 ± 0.31 ^a^	97.61 ± 1.26 ^c^	5.42 ± 0.86 ^a^	72.18 ± 3.70 ^b^	2.01 ± 0.05 ^a^

Different superscript lowercase letters indicate significant differences in different processing data. The results are shown as the mean (±SD). MC: soil moisture content; SOC: soil organic carbon; TN: soil total nitrogen; TP: soil total phosphorus; TK: soil total potassium; AN: soil available nitrogen; AP: soil available phosphorus; AK: soil available potassium. Continuous reclamation of grassland (RG), abandoned grassland (AG), and natural grazing grassland (GG).

**Table 3:** Two-factor variance analysis of soil layers on soil physical and chemical properties under land-use types.

Index	Land-Use Types		Soil Depth		Land-Use Types × Soil Depth	
	F	*p*	F	*p*	F	*p*
pH	81.41	<0.001	1410.98	<0.001	102.74	<0.001
EC (mS·cm^−3^)	224.56	<0.001	1318.95	<0.001	39.19	<0.001
MC (%)	1153.61	<0.001	1937.13	<0.001	149.81	<0.001
SOC (g·kg^−1^)	1100.24	<0.001	1539.62	<0.001	53.36	<0.001
TN (g·kg^−1^)	13.42	<0.001	9.44	0.003	1.81	0.175
TP (g·kg^−1^)	9.40	<0.001	139.08	<0.001	31.87	<0.001
TK (g·kg^−1^)	327.18	<0.001	137.18	<0.001	73.30	<0.001
AN (mg·kg^−1^)	709.88	<0.001	1979.49	<0.001	33.54	<0.001
AP (mg·kg^−1^)	176.52	<0.001	1614.36	<0.001	116.79	<0.001
AK (mg·kg^−1^)	273.25	<0.001	3275.67	<0.001	397.00	<0.001
TOC/TN	259.10	<0.001	502.09	<0.001	95.34	<0.001

MC: soil moisture content; SOC: soil organic carbon; TN: soil total nitrogen; TP: soil total phosphorus; TK: soil total potassium; AN: soil available nitrogen; AP: soil available phosphorus; AK: soil available potassium.

## Data Availability

The original data presented in the study are openly available in FigShare at 10.6084/m9.figshare.28015151.
